# Physician Characteristics Associated With Choosing Pediatrics in Japan: A Nationwide Survey

**DOI:** 10.1111/ped.70475

**Published:** 2026-07-13

**Authors:** Tomoe Otsuka, Kota Sakaguchi, Nobuyuki Ueno, Takashi Watari

**Affiliations:** ^1^ Department of Pediatrics Shimane Prefectural Central Hospital Izumo Japan; ^2^ General Medicine Center Shimane University Hospital Izumo Japan; ^3^ Shimaneken Saiseikai Gotsu General Hospital Gotsu Japan; ^4^ Integrated Clinical Education Center Kyoto University Hospital Kyoto Japan

**Keywords:** background factors, career choice, Japan, nationwide survey, pediatricians

## Abstract

**Background:**

Identifying factors related to physicians' specialty choice is crucial to ensure a sustainable healthcare workforce. However, knowledge regarding the factors influencing the choice of pediatrics remains inadequate. This study aimed to clarify physicians' background characteristics associated with choosing pediatrics and factors associated with hospital‐based practice among pediatricians in Japan.

**Methods:**

This cross‐sectional study, comprising an online survey of eligible physicians (*N* = 6415), was conducted in June 2022 via the Nikkei Medical Online website. The primary outcome was the choice of pediatrics, whereas factors associated with hospital‐based pediatricians were examined in a secondary analysis. Multivariate logistic regression analyses were used to assess the association between physician background characteristics and outcomes.

**Results:**

Among 6415 physicians, 340 (5.3%) were pediatricians. Factors associated with choosing pediatrics included being female (adjusted odds ratio [aOR] 1.84, 95% CI 1.39–2.44), having children (aOR 1.71, 95% CI 1.27–2.29), and graduating from a public university (aOR 1.67, 95% CI 1.24–2.26). Analyses restricted to pediatricians demonstrated that willingness to work in rural areas (aOR 3.24, 95% CI 1.05–10.04) was positively associated with hospital‐based practice, whereas age (per 1‐year increase; aOR 0.93, 95% CI 0.90–0.95), an urban hometown (aOR 0.54, 95% CI 0.29–0.98), and having a physician father (aOR 0.40, 95% CI 0.19–0.83) were negatively associated.

**Conclusion:**

These findings indicate that pediatric healthcare workforces may be shaped by structural factors, such as educational environment, regional background, and family background, in addition to individual preferences.

## 1 Introduction

Understanding the factors that influence physicians' specialty choices is a critical area directly linked to maintaining high‐quality healthcare delivery systems, correcting regional disparities, and addressing future healthcare needs [1]. Therefore, it is essential to clarify the factors that shape physicians' career trajectories [2, 3].

Previous studies have examined the factors characterizing physicians' specialty choices, including personal attributes such as sex and age, as well as socioeconomic status, such as parental occupation, household income, and educational background [3, 4]. For example, having parents who work in the healthcare profession has been associated with academic achievements and better opportunities to enter a medical school. These factors may subsequently shape career pathways [5]. Furthermore, higher family socioeconomic status has been suggested to influence postgraduate career paths and specialty selection [4]. Additionally, the place of origin and regional healthcare experiences during student years may influence future practice locations and inclination toward primary care [6, 7, 8]. These studies demonstrate that physicians' specialty choices are molded by complex factors encompassing not only personal values, but also the family environment and place of upbringing. However, these findings may not apply to all medical fields. This is because patient demographics, work environments, and required expertise vary significantly across specialties. In pediatrics, in particular, clinical care affects not only the patients themselves but also their families and the social setting [9]. These clinical characteristics are associated with physicians' preferences and may influence their specialty choices. Notably, pediatricians comprise a significantly higher proportion of female physicians [10], value work–life balance, and show a greater degree of empathy toward patients [11, 12, 13]. Thus, pediatrics involves unique inherent characteristics. Consequently, whether the factors influencing specialty choices identified in previous studies also apply to pediatrics remains unclear.

Furthermore, in Japan's pediatric healthcare setting, maintaining a sufficient number of not only pediatricians but also hospital‐based physicians is a critical challenge [14]. The pediatric healthcare system is increasingly centralized to improve the efficiency of medical resources [15]. In particular, functions such as emergency care, inpatient care, and the management of critically ill patients are concentrated in local core hospitals. Consequently, there is a need to ensure a stable supply of hospital‐based physicians to provide these services. However, shortages of hospital‐based physicians have been a major concern, with regional healthcare provision systems remaining fragile [16, 17]. Pediatricians' career trajectories vary, but hospital‐based physicians and clinic‐based pediatricians represent the core. To understand the structure of the healthcare delivery system, comparing the types of practices and clarifying the characteristics of physicians driving the choice to work in hospitals is essential. However, research focusing on the factors influencing these work‐style choices remains limited.

Therefore, the primary aim of this study was to clarify the background factors associated with the choice of pediatrics. Secondarily, we examined the factors associated with being hospital‐based pediatricians.

## 2 Methods

### 2.1 Study Design and Setting

This study is a nationwide cross‐sectional study conducted in June 2022. The survey was conducted through “Nikkei Medical Online” [18], one of Japan's largest portals for physicians. At the time of the survey, approximately 200,299 physicians were registered. The survey was posted on the website, and physicians accessed and completed it voluntarily. As an incentive, participants were awarded 10 points (equivalent to less than $0.50), which could be exchanged online for small items. This study was conducted in accordance with the STROBE (Strengthening the Reporting of Observational Studies in Epidemiology) guidelines.

### 2.2 Participants

The physicians who completed the survey held a Japanese medical license. To ensure data reliability and validity, participants meeting any of the following criteria were excluded: (1) inconsistent responses such as discrepancy between age and years since graduation (*n* = 270), (2) missing data in key variables (*n* = 9), (3) sex reported as “other” or missing (*n* = 58), and (4) responses indicating “other/unclassifiable” for the number of beds in the affiliated facility (*n* = 295).

**FIGURE 1:**
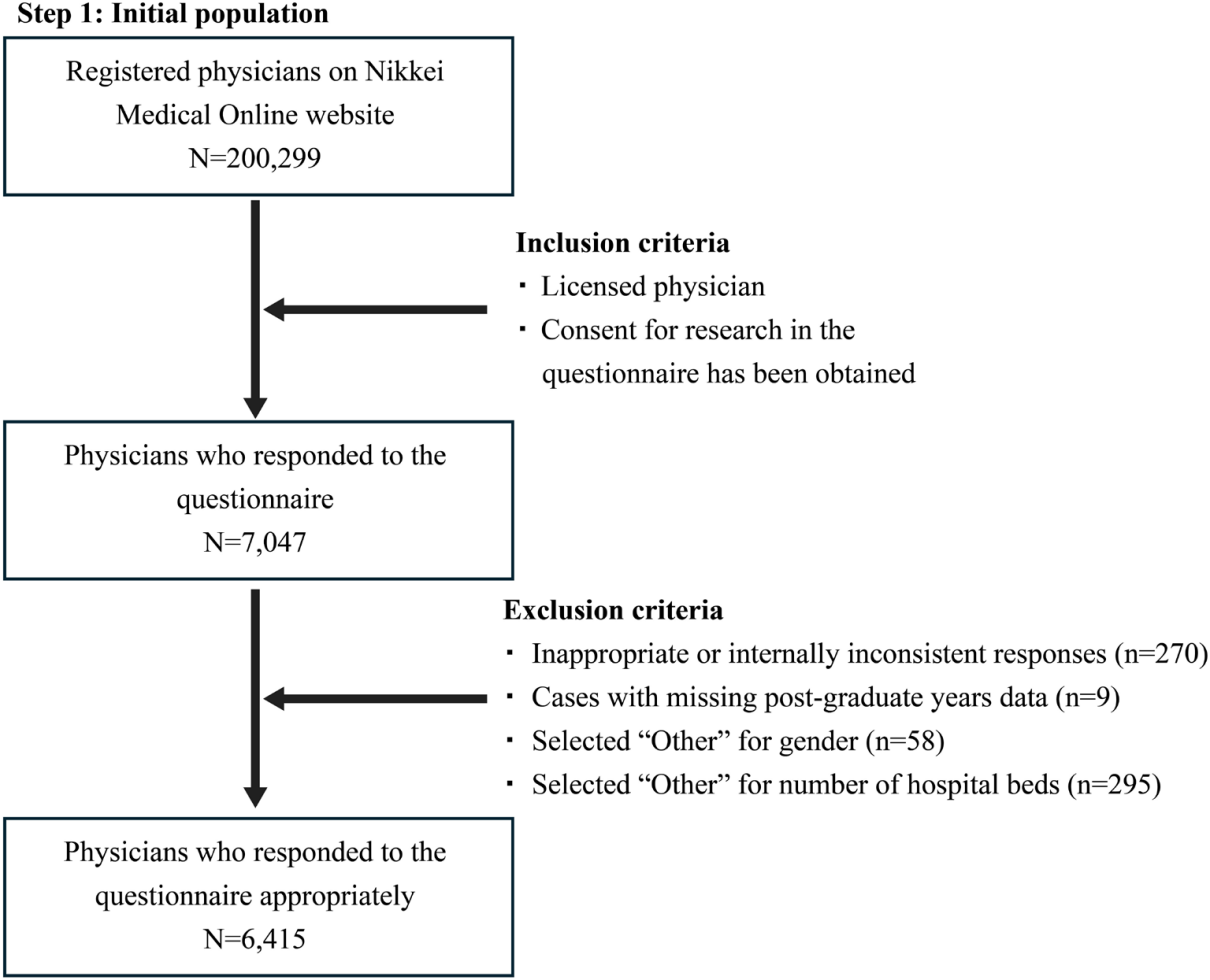
Flowchart demonstrating participant inclusion and exclusion. Flowchart showing the inclusion and exclusion criteria of physicians in the nationwide cross‐sectional survey conducted in June 2022. Of the 200,299 registered physicians, 7047 completed the survey. After applying exclusion criteria (inappropriate or internally inconsistent responses, cases with missing postgraduate years data, selected “Other” for gender, selected “Other” for number of hospital beds), 6415 physicians were included in the final analysis.

After applying the exclusion criteria, 6415 physicians were enrolled in the final analyses from the initial sample of 7047 participants (Figure 1).

### 2.3 Variable Definitions and Measurements

The primary outcome of this study was the selection of pediatrics as a specialty. Based on the participants' self‐reported specialties, physicians were categorized as pediatricians or non‐pediatricians. For the secondary analysis of the pediatricians, practice setting was classified according to the number of beds in the affiliated medical institutions. According to the Japanese Medical Care Act classification, physicians working in facilities with 20 or more beds are defined as hospital‐based pediatricians, whereas those working in facilities with 19 or fewer beds are defined as clinic‐based pediatricians.

Covariates included demographic and background characteristics. Age (continuous) and sex (male or female) were the basic demographic variables. Family background variables included having children (yes/no) and the parental occupation. Based on previous studies, whether the father or mother was a physician was defined as a binary variable (physician vs. non‐physician). Educational and geographic background variables comprised the type of medical school and place of origin. Medical schools were categorized as public (including Jichi Medical University and the National Defense Medical College) or private universities. Hometown was defined as the region where the participant resided for the longest period prior to entering medical school. Tokyo's 23 wards and government‐designated cities were classified as urban areas, whereas all other areas were considered rural. Regarding career orientation, we questioned the participants' willingness to work in rural areas. Those who responded “currently working” or “willing to work in the future” were classified as “willing,” whereas others were classified as “unwilling.” The presence of obligations toward rural practice was also included as a covariate. Facility bed capacity was categorized into four groups: ≤ 19 beds, 20–99 beds, 100–299 beds, and ≥ 300 beds.

### 2.4 Statistical Analysis

The baseline characteristics of the participants were summarized using descriptive statistics. Continuous variables are presented as mean ± standard deviation, whereas categorical variables are presented as counts and percentages. Comparisons between pediatricians and non‐pediatricians, and between hospital‐based and clinic‐based pediatricians, were performed using Student's *t*‐test for continuous variables and chi‐square test for categorical variables. To identify independent factors associated with specialty choice and practice setting, multivariable logistic regression analyses were performed. In the first model, all physicians were included, with the outcome being the choice of pediatrics. In the second model, only pediatricians were included, with the outcome variable being a hospital‐based pediatrician. The following variables were included as explanatory variables in the models: age, sex, having children, father's occupation, mother's occupation, type of medical school, hometown, willingness to work in rural areas, and obligation toward rural practice. Although “years since graduation”—a variable strongly correlated with age—was included in the univariate analysis, it was excluded from the multivariate analysis to avoid multicollinearity. In addition, because “parental household income” had many missing values and raised concerns about selection bias, it was excluded from the analysis to maintain the model's validity and statistical power. Multicollinearity was assessed using the variance inflation factor (VIF), and all variables showed VIF values < 1.3, indicating no significant multicollinearity. Model fit was evaluated using the Hosmer–Lemeshow test and c‐statistic (area under the curve). All statistical tests were two‐sided, with *p* < 0.05 being considered statistically significant. All analyses were performed using Stata version 17 software (StataCorp, College Station, TX, USA).

### 2.5 Ethical Considerations

This study was approved by the Ethics Committee of Shimane University, Faculty of Medicine (approval number: 20211124‐1). The participants were informed of the purpose and content of the study on the web survey platform. Informed consent was obtained electronically by clicking a consent button prior to participation. This study was conducted in accordance with the principles of the Declaration of Helsinki.

## 3 Results

### 3.1 Participant Characteristics

Table 1 shows the background characteristics of the 6415 physicians included in the analysis. There were 340 pediatricians (5.3%) and 6075 non‐pediatricians (94.7%). The mean age was 47.4 ± 12.3 years for pediatricians and 47.8 ± 13.0 years for non‐pediatricians; no significant difference was observed between the two groups (*p* = 0.572). The proportion of female pediatricians was significantly higher than that of non‐pediatricians (21.8% vs. 14.7%, *p* < 0.001). The proportion of those with children was also significantly higher among pediatricians than among non‐pediatricians (78.5% vs. 72.1%, *p* = 0.010). Educational background survey showed that the proportion of pediatricians graduating from public universities was significantly higher than that of non‐pediatricians (83.2% vs. 73.8%, *p* < 0.001). Regarding workplace characteristics, pediatricians were more likely to work in large hospitals with 300 or more beds (59.4% vs. 49.3%, *p* = 0.001). Conversely, the proportion expressing a willingness to work in rural areas was significantly lower among pediatricians than among non‐pediatricians (14.7% vs. 19.9%, *p* = 0.018). There were no significant differences between the groups in terms of hometown, the proportion of parents who were physicians, or obligation for rural practice.

**TABLE 1:** Demographic characteristics of the pediatricians and non‐pediatricians.

Variable	Pediatricians (*n* = 340)	Non‐pediatricians (*n* = 6075)	*p*
Age (years)	47.4 ± 12.3	47.8 ± 13.0	0.572
Postgraduate years	21.5 ± 11.7	21.5 ± 12.4	0.933
Sex, *n* (%)			**< 0.001**
Male	266 (78.2)	5183 (85.3)	
Female	74 (21.8)	892 (14.7)	
Having children, *n* (%)			**0.010**
Yes	267 (78.5)	4379 (72.1)	
No	73 (21.5)	1696 (27.9)	
Father is a physician, *n* (%)	76 (22.4)	1638 (27.0)	0.062
Mother is a physician, *n* (%)	8 (2.4)	272 (4.5)	0.062
Type of medical school, *n* (%)			**< 0.001**
Public or other	283 (83.2)	4484 (73.8)	
Private	57 (16.8)	1591 (26.2)	
Hometown, *n* (%)			0.513
Urban	122 (35.9)	2287 (37.7)	
Rural	218 (64.1)	3788 (62.4)	
Willingness to work in rural areas, *n* (%)	50 (14.7)	1211 (19.9)	**0.018**
Obligation for rural practice, *n* (%)	75 (22.1)	1377 (22.7)	0.794
Facility bed capacity, *n* (%)			**0.001**
≤ 19 beds (Clinics)	68 (20.0)	1299 (21.4)	
20–99 beds	11 (3.2)	359 (5.9)	
100–299 beds	59 (17.4)	1422 (23.4)	
≥ 300 beds	202 (59.4)	2995 (49.3)	

*Note:* Data are presented as mean ± standard deviation or number (%). The bold values in the tables indicate statistical significance (*p* < 0.05).

### 3.2 Factors Associated With Choosing Pediatrics

Table 2 shows the results of the multivariate logistic regression analysis exploring the independent factors associated with the choice of pediatrics. Female physicians were significantly more likely to choose pediatrics than male physicians (adjusted odds ratio [aOR] 1.84, 95% CI 1.39–2.44, *p* < 0.001). Physicians with children were also more likely to choose pediatrics compared with those without children (aOR 1.71, 95% CI 1.27–2.29, *p* < 0.001). Furthermore, graduates from public universities were significantly more likely to choose pediatrics than those from private universities (aOR 1.67, 95% CI 1.24–2.26, *p* = 0.001). Although the univariate analysis showed differences in the willingness to work in rural areas and facility bed capacity, the multivariate analysis did not reveal any significant associations. Similarly, the proportion of parents who were physicians, hometown, and obligation for rural practice were not significantly associated with choosing pediatrics.

**TABLE 2:** Factors associated with choosing pediatrics (multivariate logistic regression analysis).

Variable	Adjusted odds ratio	95% CI	*p*
Sex			
Male	1.00 (Ref)		
Female	1.84	1.39–2.44	**< 0.001**
Age (per 1‐year increase)	1.00	0.99–1.01	0.741
Having children			
No	1.00 (Ref)		
Yes	1.71	1.27–2.29	**< 0.001**
Father is a physician			
No	1.00 (Ref)		
Yes	0.93	0.70–1.23	0.592
Mother is a physician			
No	1.00 (Ref)		
Yes	0.60	0.28–1.25	0.172
Type of medical school			
Private	1.00 (Ref)		
Public or other	1.67	1.24–2.26	**0.001**
Hometown			
Rural	1.00 (Ref)		
Urban	0.94	0.75–1.19	0.610
Obligation for rural practice			
No	1.00 (Ref)		
Yes	1.05	0.80–1.38	0.710
Willingness to work in rural areas			
No	1.00 (Ref)		
Yes	0.75	0.55–1.03	0.074
Facility bed capacity			
≤ 19 beds (Clinics)	1.00 (Ref)		
20–99 beds	0.58	0.30–1.11	0.098
100–299 beds	0.76	0.53–1.10	0.145
≥ 300 beds	1.28	0.94–1.74	0.118
Reference	1.00 (Ref)		

*Note:* Data were analyzed using multivariate logistic regression analysis (*n* = 6415). The bold values in the tables indicate statistical significance (*p* < 0.05).

Abbreviations: CI, confidence interval; Ref, reference group.

### 3.3 Comparison Between Hospital‐Based and Clinic‐Based Pediatricians

Table 3 compares the backgrounds of hospital‐based physicians (≥ 20 beds) and clinic‐based physicians (< 20 beds) among the 340 pediatricians. Participants consisted of 272 hospital‐based physicians (80.0%) and 68 clinic‐based physicians (20.0%). Hospital‐based pediatricians were significantly younger than clinic‐based pediatricians (45.4 ± 11.7 vs. 55.0 ± 11.7 years, *p* < 0.001) and had fewer years of experience since graduation (19.8 ± 11.3 vs. 28.2 ± 11.0 years, *p* < 0.001). The proportion of physicians whose fathers were doctors was significantly lower among hospital‐based physicians than clinic‐based physicians (19.5% vs. 33.8%, *p* = 0.011). The proportion of physicians from rural areas was significantly higher among hospital‐based physicians than among clinic‐based physicians (67.6% vs. 50.0%, *p* = 0.007). Furthermore, the proportion expressing a willingness to work in rural areas was significantly higher among hospital‐based physicians than among clinic‐based physicians (16.9% vs. 5.9%, *p* = 0.022). No significant differences were observed by practice type regarding sex, having children, the proportion of mothers who are doctors, medical school attendance, or the existence of an obligation for rural practice.

**TABLE 3:** Characteristics of pediatricians based on their practice setting.

Variable	Hospital‐based (*n* = 272)	Clinic‐based (*n* = 68)	*p*
Age (years)	45.4 ± 11.7	55.0 ± 11.7	**< 0.001**
Postgraduate years	19.8 ± 11.3	28.2 ± 11.0	**< 0.001**
Sex, *n* (%)			0.693
Male	214 (78.7)	52 (76.5)	
Female	58 (21.3)	16 (23.5)	
Having children, *n* (%)	210 (77.2)	57 (83.8)	0.235
Father is a physician, *n* (%)	53 (19.5)	23 (33.8)	**0.011**
Mother is a physician, *n* (%)	6 (2.2)	2 (2.9)	0.721
Type of medical school, *n* (%)			**0.042**
Public or other	232 (85.3)	51 (75.0)	
Private	40 (14.7)	17 (25.0)	
Hometown, *n* (%)			**0.007**
Urban	88 (32.4)	34 (50.0)	
Rural	184 (67.6)	34 (50.0)	
Willingness to work in rural areas, *n* (%)	46 (16.9)	4 (5.9)	**0.022**
Obligation for rural practice, *n* (%)	62 (22.8)	13 (19.1)	0.513

*Note:* Data are presented as mean ± standard deviation or number (%). The bold values in the tables indicate statistical significance (*p* < 0.05).

### 3.4 Factors Associated With Hospital‐Based Practice Among Pediatricians

Table 4 shows the results of multivariate logistic regression analysis examining the factors associated with hospital‐based practices among pediatricians. Age showed a negative association with hospital‐based practice; for each additional year of age, the odds of being a hospital‐based physician decreased significantly (aOR 0.93, 95% CI 0.90–0.95, *p* < 0.001). Physicians expressing a willingness to work in rural areas had significantly higher odds of being hospital‐based physicians compared to those without such willingness (aOR 3.24, 95% CI 1.05–10.04, *p* = 0.041). Physicians from urban areas had significantly lower odds of being a hospital‐based physician compared to those from rural areas (aOR 0.54, 95% CI 0.29–0.98, *p* = 0.042). Furthermore, having a physician father was significantly associated with lower odds of being a hospital‐based physician (aOR 0.40, 95% CI 0.19–0.83, *p* = 0.014). In contrast, sex, having children, obligation for rural practice, type of medical school, and the proportion of mothers who are physicians were not significantly associated with being a hospital‐based physician.

**TABLE 4:** Factors associated with continuing hospital‐based practice among pediatricians.

Variable	Adjusted odds ratio	95% CI	*p*
Sex			
Male	1.00 (Ref)		
Female	0.65	0.31–1.37	0.253
Age (per 1‐year increase)	0.93	0.90–0.95	**< 0.001**
Having children			
No	1.00 (Ref)		
Yes	0.94	0.41–2.14	0.885
Willingness to work in rural areas			
No	1.00 (Ref)		
Yes	3.24	1.05–10.04	**0.041**
Obligation for rural practice			
No	1.00 (Ref)		
Yes	0.94	0.43–2.03	0.867
Type of medical school			
Private	1.00 (Ref)		
Public or other	1.58	0.73–3.45	0.246
Hometown			
Rural	1.00 (Ref)		
Urban	0.54	0.29–0.98	**0.042**
Father is a physician			
No	1.00 (Ref)		
Yes	0.40	0.19–0.83	**0.014**
Mother is a physician			
No	1.00 (Ref)		
Yes	1.02	0.17–6.24	0.985
Reference	1.00 (Ref)		

*Note:* Data were analyzed using multivariate logistic regression analysis (*n* = 340). Hospital‐based practice was defined as 1, and clinic‐based practice (including ownership) was defined as 0. No multicollinearity was detected (variance inflation factor < 1.30). The bold values in the tables indicate statistical significance (*p* < 0.05).

Abbreviations: CI, confidence interval; Ref, reference group.

## 4 Discussion

### 4.1 Principal Findings

In this nationwide cross‐sectional study of physicians in Japan, we comprehensively examined factors associated with choosing pediatrics as a specialty and factors associated with choosing hospital‐based practice among pediatricians. Multivariate analysis revealed that being female, having children, and graduating from a public university were independently associated with choosing pediatrics. Furthermore, in an analysis focused on pediatricians, being young, expressing a willingness to work in rural areas, belonging to a rural background, and having a father who was not a physician were independently associated with choosing hospital‐based practice. These findings indicate that the choice of pediatrics as a specialty is driven by multiple factors, including an individual's life course, inherent values, educational background, and regional orientation. Furthermore, they suggested that career stage, family background, and orientation toward community‐based healthcare may influence pediatricians' choice of practice settings.

### 4.2 Factors Associated With Choosing Pediatrics

In this study, females were significantly more likely than males to choose pediatrics. Pediatrics is traditionally known as a specialty representing a high number of female physicians [4, 14, 19]. Multiple factors have been suggested to contribute to this trend, including a high level of interest in the pediatric patient population [19], the presence of role models, and perceptions regarding work–life flexibility [19, 20]. These differences may have been reflected in the career choices made by male and female physicians.

Moreover, the higher proportion of pediatricians with children may reflect the relationship between specialty choice and family life. Prior studies have reported that flexibility in working hours and work–life balance are key factors influencing the choice of specialty, and our results are consistent with these findings [21]. However, although the difference in the proportion of physicians with children between pediatricians and non‐pediatricians was statistically significant, the absolute difference was small. Therefore, its practical impact should be interpreted with caution. In Japan, female physicians are more likely to leave their positions during pregnancy and childbirth [22]. Particularly in pediatrics, with a higher proportion of female physicians, the need to improve the working environment to enable career continuity has been increasingly emphasized [23]. Flexible working arrangements, such as reduced working hours and exemptions from night shifts, have been increasingly introduced in pediatric departments [23]. Consequently, a support system for returning to work has been established, and role models are now clearly visible. Such environments may influence preferences for balancing childcare and career and may also be associated with the choice of pediatrics. However, since this study is cross‐sectional, a causal relationship between specialty choice and the presence of children cannot be established. Therefore, these results do not indicate a causal relationship and should be interpreted in the context of other background factors.

Graduation from a public university was independently associated with the pediatric choice. Previous studies on specialty choice have primarily focused on individual characteristics and lifestyle preferences [4, 20], whereas the influence of the educational environment has received less attention. Our findings suggest that the institutional characteristics of medical schools may influence specialty choices. In Japan, public university medical schools tend to focus on collaboration with community healthcare. Consequently, opportunities for community‐based medical education are more frequently available than those for private universities [24]. Community‐based medical education emphasizes healthcare that considers social needs and patients' life backgrounds [25]. Such educational concepts can influence students' professional identity formation [26]. Previous studies have also reported that physicians admitted through region‐oriented admission programs and those with an obligation for rural practice are more likely to choose pediatrics [27], and our results are consistent with these findings. In contrast, differences in university type may reflect differences in student backgrounds to some extent. These differences are shaped not only by variations in educational content but also by differences in socioeconomic background, childhood experiences, and the characteristics emphasized in the admissions process. Therefore, the results of this study suggest that, in addition to the educational environment, factors such as family background, childhood experiences, and the characteristics of the admissions process are involved in complex relationships.

These findings suggest that the choice of pediatrics may involve a complex interaction of personal factors, such as sex and family background, along with social factors, including the educational environment. The results of this study indicate that factors identified in previous research also apply to the choice of pediatrics. Furthermore, the findings suggest the possibility of factors specific to pediatrics, such as educational background.

### 4.3 Factors Associated With Hospital‐Based Practice Among Pediatricians

To the best of our knowledge, previous studies have not examined the factors influencing the choice of practice type. This study is unique in that it identified the background factors associated with the choice of a hospital‐based practice.

First, the higher proportion of younger physicians among hospital‐based practitioners may reflect their career development trends. Since training to become a pediatric specialist and building clinical experience primarily take place in hospitals, choosing hospital work early in one's career is common. In Japan, the average age of clinic‐based physicians is higher than that of hospital‐based physicians [28]; our findings likely reflect this established career trajectory for pediatricians.

Second, physicians willing to work in rural areas and those with rural backgrounds were more likely to work in hospitals. In Japan, a regional imbalance exists in the distribution of physicians, with physicians concentrated in urban and rural areas having limited medical resources [16, 17]. In rural areas, local core hospitals provide pediatric care, including inpatient care and emergency services [29]. Therefore, hospital‐based practice is a reasonable choice for physicians willing to work in rural areas. Furthermore, previous research indicates that individuals from rural areas tend to work in rural areas later in life [7, 30], suggesting that a rural background may be associated with rural orientation. Considering the above discussion, the desire to work in rural areas and rural background may reflect the structural characteristics of pediatric healthcare delivery systems in rural areas. However, since clinic‐based physicians often have permanent practice locations, this difference does not necessarily reflect differences in preferences. Such limitations may have influenced the responses, and the results of this study should be interpreted with caution.

Third, the finding that having a father who is not a physician is associated with hospital‐based practice may be influenced by the option of continuing the family business. However, this study does not categorize fathers by their practice type (hospital‐based or clinic‐based practice); therefore, the results should be interpreted with caution. Previous studies have reported that the medical profession exhibits a tendency toward generational reproduction, with the children of physicians being more likely to enter the medical profession [5, 31]. In Japan, many clinics are maintained through family transitions, and for physicians from medical families, taking over a clinic is a real career option [32]. In contrast, physicians without such family backgrounds may be less likely to have opportunities for clinic inheritance; therefore, they may be more likely to pursue hospital‐based careers. Our findings suggest that family background plays a role in shaping physicians' choices in practice settings.

These findings suggest that the choice of hospital‐based practice is associated not only with differences in practice setting but also with multiple factors, including career stage, regional orientation, and family background.

### 4.4 Implications and Strengths

The main strength of this study is that it used nationwide physician data to quantitatively examine the factors associated with specialty choice and practice setting among pediatricians. In particular, the finding that graduation from a public university was independently associated with choosing pediatrics suggests a potential influence of the educational environment on specialty orientation, whereas other factors, such as admissions policies and students' backgrounds, cannot be ruled out. In addition, the finding that choosing hospital‐based practice among pediatricians was significantly associated with rural background and willingness to work in rural areas provides necessary evidence of specialty choice and practice setting being related to the educational environment and regional background. Furthermore, the negative association between hospital‐based practice and having a physician father highlights the potential influence of intergenerational factors, such as family clinic succession. These findings offer a new perspective for understanding how structural factors, including educational systems, regional background, and family structure, may shape the pediatric workforce. These findings suggest that policies to secure pediatricians should not focus solely on increasing the number of physicians but also consider structural factors such as educational systems, regional orientation, and family background when formulating strategies for workforce development and placement. To ensure the sustainability of pediatric healthcare delivery, comprehensive policy approaches that address specialty choices and practical settings are necessary.

### 4.5 Limitations

This study has some limitations. First, as this was a cross‐sectional study, causality could not be established. The associations shown in this study may reflect the outcomes of the decision‐making process rather than direct influences on career selection. Second, this study used self‐reported data from a web‐based survey, which may have been subject to participant and selection biases. Specifically, there may be a high proportion of physicians who frequently use medical information websites or who have a strong interest in their careers, which may contribute to the strong correlations with specialty choice and regional preferences observed. In addition, a small incentive was provided for responses, which may have influenced participation. Consequently, these factors may have limited the generalizability of our findings to all physicians in Japan. Third, when comparing willingness to work in rural areas, caution is required in interpreting the results because opportunities to choose work location differ depending on the practice type. Fourth, unmeasured confounding factors that could not be captured in this study may exist, such as income levels, the influence of mentor physicians, the culture of each specialty, and the educational content of each university, which may influence specialty choice and practice setting. These findings demonstrate that the associations and causal interpretations should be interpreted cautiously. Future research that includes more detailed background information is required to further examine the mechanisms underlying career‐building processes.

## 5 Conclusion

Overall, the study findings suggest that the paediatric workforce may be shaped not only by individual preferences but also by structural factors such as educational environments, regional background, and family characteristics. Further research is warranted to better describe how these factors influence physicians' career decisions, and how they may inform workforce policies aimed at sustaining paediatric healthcare systems.

## Author Contributions

T.O. conceptualized and designed the study. K.S. acquired the data. K.S. performed statistical analyses and drafted the manuscript. T.W. and N.U. critically reviewed the manuscript for intellectual content. All the authors have read and approved the final version of the manuscript.

## Funding

This study was supported by the National Academic Research Grant Fund (JSPS KAKENHI, 20H03913).

## Conflicts of Interest

The authors declare no conflicts of interest.

## Data Availability

The data supporting the findings of this study are available from the General Medicine Center of Shimane University Hospital.
